# 184. Implications of *Enterococcus faecalis* penicillin susceptibility in patients with bacteremia

**DOI:** 10.1093/ofid/ofad500.257

**Published:** 2023-11-27

**Authors:** Jason Hedvat, Melissa R Gitman, Angella Nwoye, Li Chen, Colleen Martin, Mandie Wang, Chirag Vasa, Jaclyn A Cusumano

**Affiliations:** Mount Sinai Beth Israel, New York, New York; Icahn School of Medicine at Mount Sinai, New York, NY; Long Island University, Brooklyn, New York; Long Island University, Brooklyn, New York; Long Island University, Brooklyn, New York; Long Island University, Brooklyn, New York; Mount Sinai Queens, Astoria, New York; Long Island University, Brooklyn, New York

## Abstract

**Background:**

*Enterococcus faecalis* isolates with an elevated penicillin minimum inhibitory concentration (MIC) ≥ 4 mg/L have decreased *in vitro* ampicillin plus ceftriaxone bactericidal and synergistic activity, despite ampicillin susceptibility. This phenotype’s implication on patient outcomes and prevalence in the US is unknown due to limited penicillin susceptibility reporting.

**Methods:**

This was a retrospective cohort study of adult patients with *E. faecalis* bacteremia between August 20, 2018 and May 13, 2021 from the Mount Sinai Health System in New York City. Patients were excluded if they were discharged or deceased within 48 hours of index blood culture, did not receive active definitive therapy, or the blood culture was determined to be contamination. The primary outcome was differences in 30-day and 90-day mortality based on the penicillin MIC and the secondary outcome was microbiological relapse. Multivariable logistic regression was used to identify risk factors for 90-day mortality. Corresponding blood isolates were tested for penicillin susceptibility by broth microdilution in accordance with CLSI.

**Results:**

A total of 124 patients were included, with 27% having an elevated penicillin MIC ≥ 4 mg/L. Differences in baseline characteristics (Table 1) as well as source of infection and treatments received (Table 2) were similar between groups. Endocarditis infected 18.5% of patients and was less common in cases with an elevated penicillin MIC. Thirty-day and 90-day mortality, and microbiological relapse were higher in cases with an elevated penicillin MIC (14.4% vs. 20.6%, p=0.58; 20% vs. 26.5%, p=0.59; and 4.4% vs. 8.8%, p=0.39, respectively). Pitt bacteremia score and prior ceftriaxone usage had a higher odds of 90-day mortality (OR 1.2 [95% CI 1.03-1.4], p=0.02; OR 2.8 [95% CI 1.02-7.9], p=0.04, respectively), whereas definitive treatment with an aminopenicillin compared to piperacillin/tazobactam was protective of 90-day mortality (OR 0.21 [95% CI 0.05-0.93], p=0.04).

**Figure:**
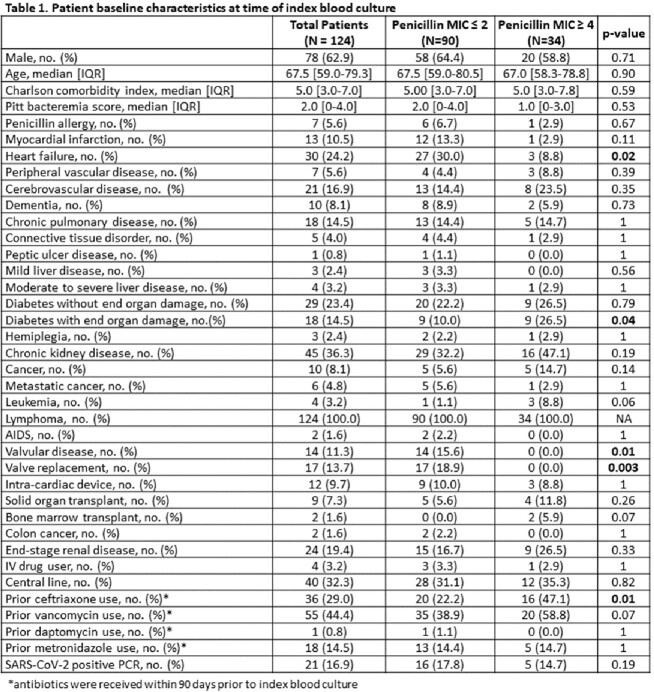

**Figure:**
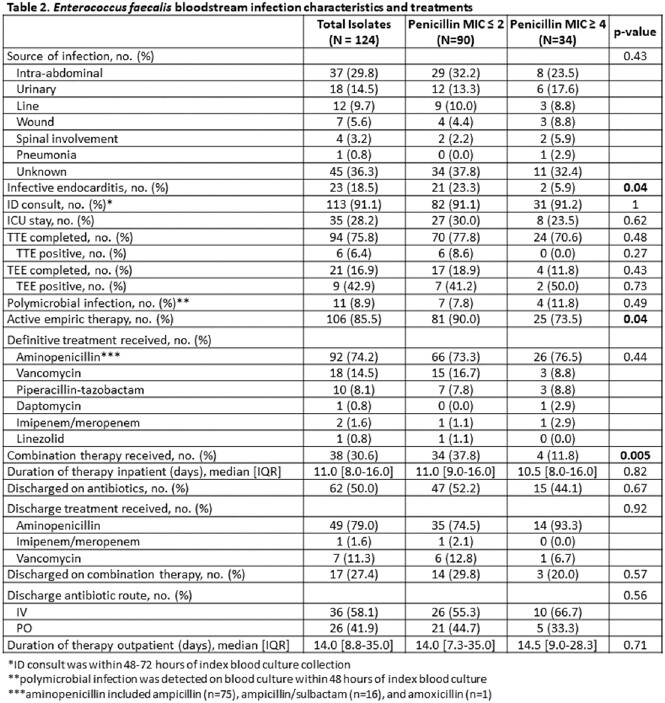

**Conclusion:**

*E. faecalis* with an elevated penicillin MIC ≥ 4 mg/L is present in over a quarter of patients across New York City. Larger studies are warranted to determine the impact on patient outcomes. *E. faecalis* bacteremia treatment with piperacillin/tazobactam may be associated higher mortality.

**Disclosures:**

**Jaclyn A. Cusumano, PharmD, BCIDP**, Shionogi: Advisor/Consultant

